# Governance of Clinical AI applications to facilitate safe and equitable deployment in a large health system: Key elements and early successes

**DOI:** 10.3389/fdgth.2022.931439

**Published:** 2022-08-24

**Authors:** Frank Liao, Sabrina Adelaine, Majid Afshar, Brian W. Patterson

**Affiliations:** ^1^ BerbeeWalsh Department of Emergency Medicine, UW-Madison, Madison, WI, United States; ^2^ Department of Information Services, UW Health, Madison, WI, United States; ^3^ Department of Medicine, UW-Madison, Madison, WI, United States; ^4^ Department of Biostatistics and Medical Informatics, UW-Madison, Madison, WI, United States; ^5^ Department of Industrial and Systems Engineering, UW-Madison, Madison, WI, United States

**Keywords:** Clinical AI, AI, AI adoption, predictive analytics, governance, oversight, ethics, equity

## Abstract

One of the key challenges in successful deployment and meaningful adoption of AI in healthcare is health system-level governance of AI applications. Such governance is critical not only for patient safety and accountability by a health system, but to foster clinician trust to improve adoption and facilitate meaningful health outcomes. In this case study, we describe the development of such a governance structure at University of Wisconsin Health (UWH) that provides oversight of AI applications from assessment of validity and user acceptability through safe deployment with continuous monitoring for effectiveness. Our structure leverages a multi-disciplinary steering committee along with project specific sub-committees. Members of the committee formulate a multi-stakeholder perspective spanning informatics, data science, clinical operations, ethics, and equity. Our structure includes guiding principles that provide tangible parameters for endorsement of both initial deployment and ongoing usage of AI applications. The committee is tasked with ensuring principles of interpretability, accuracy, and fairness across all applications. To operationalize these principles, we provide a value stream to apply the principles of AI governance at different stages of clinical implementation. This structure has enabled effective clinical adoption of AI applications. Effective governance has provided several outcomes: (1) a clear and institutional structure for oversight and endorsement; (2) a path towards successful deployment that encompasses technologic, clinical, and operational, considerations; (3) a process for ongoing monitoring to ensure the solution remains acceptable as clinical practice and disease prevalence evolve; (4) incorporation of guidelines for the ethical and equitable use of AI applications.

## Introduction

Artificial intelligence (AI) holds the promise to transform clinical care (1), and is increasingly being used in clinical practice. However, appropriate governance of these models remains in its infancy, especially as larger governing bodies like the Food and Drug Administration and World Health Organization are trying to keep up with the advancements in technology and its role in health care. Unlike other sectors, healthcare must carry a lower tolerance for error and bias as AI-driven tools have a direct impact on patient lives and unchecked errors may cause harm or death (2). UW Health, like many academic institutions, frequently encounters new commercial products and scientific innovations that leverage AI for healthcare delivery in both diagnostics and prognosis. While several groups have discussed the importance of, and methodologies for, responsible development of these interventions (3), the accountability of safe and effective deployment of AI-driven applications ultimately falls onto the health system. As the ethics surrounding AI-development has received increasing scrutiny (4), there has been little literature focusing on institutional governance. As we expand our technical ability to provide solutions, more skepticism and questions surface, and at times resistance, around the suitability of using AI in routine clinical care from all levels of the organization, ranging from front-line clinical staff to executive leadership. In response to these questions and the challenges for implementation, the health system recognized the need for a governance structure to endorse and oversee adoption, implementation, and ongoing value evaluation of AI-driven applications. This case study describes the development and nature of governance of clinical AI applications at our institution.

## The role of governance

### Challenges

During deployment of the first set of AI-driven applications, we encountered several challenges unique to the field. From a systems perspective these challenges can be grouped into three domains, where each domain represents a particular constituency with associated considerations (Table 1). The first domain is clinical, and its constituents are the patients, clinicians and other front-line users of the AI solution. The challenges associated with the first domain are related to clinical acceptability of the AI output, and actionability (in terms of personal agency), as well as explainability. The goal of governance for this domain is maintaining patient safety, as well as securing clinician acceptance and adoption.

**Table 1:** Challenges to the adoption of AI categorized by domains with associated constituencies and goals.

Domain	Constituents	Goals of governance
Clinical	Patients, clinicians, staff	Patient safety, model effectiveness, explainability and adoption
Operational	Clinical and operational stakeholders	Integration of AI models into routine health system operations
Leadership	Hospital and health system leaders	Endorsement by senior leadership, integration into overall strategic plans

The second domain is operational; the constituents of this domain represent the systems-level components that are part the care delivery mechanism. This group includes the stakeholders that represent clinical operations, information services and informatics. The challenges for the second domain are related to actionability (in terms of clinical protocols and governance), performance validity, sustainability, and accountability (in terms of ongoing support mechanisms). The goal of the governance for this domain is complementary oversight that is compatible with the routine operating model of the health system.

The third domain is leadership and its constituents are those who manage the strategic direction of the health system, hold key decision rights, and govern the resources. The challenges for the third domain are related to oversight, accountability, and equitability. The goal of the governance for this domain is endorsement by senior leadership in health operations.

As we operationalized predictive models, we surfaced challenges in each domain. Some domains, such as clinical and operational, required more focus earlier in development, with rapid adaption and evolution, while the leadership domain, adapted at a different rate that required more focus with commensurate experience of the organization. The focus of the domain shifted and adapted over time depending on the maturity of each domain and the individualized needs of each AI application.

To address the challenges in the first and second domain during rollout of our initial models, individual solution workgroups were established in an *ad hoc* fashion. The responsibility of these workgroups was performing due diligence and providing detailed scrutiny of the AI solutions to establish the necessary validity both clinically, technically, and ethically. Examples of specific activities include retrospective and prospective validations of the performance of the AI solution on the UWH patient population as a whole and on specific demographic sub-populations; closer examination of the clinical inputs or variables used by the AI solution; the suitability of the solution's output within its specific operational and clinical context; and ongoing assessment of the solution's performance, clinician adoption and usage, and other related metrics.

The output of the workgroups was also synthesized and disseminated to the constituents in the second domain to procure complementary governance such as the approval of new or updated clinical protocols that incorporated the AI solution, or operational buy-in to update the ongoing or routine processes of the health system. The designation of these workgroups evolved along with the AI maturity of the organization, beginning with “algorithm workgroups”, then to “algorithm committees”, with a current designation of “algorithm sub-committees”. Initially, challenges within the third domain, leadership, were addressed through executive sponsorship of a specific use case, which constituted simple endorsement for smaller applications and executive steering committees when necessary for larger projects.

The composition and membership of workgroups were multi-disciplinary, as necessary to perform their function. Key disciplines included clinical subject matter expertise, data science, informatics, information technology, clinical operations, bioethics, human factors or design thinking. Common roles that were represented included physicians, nurses, data scientists, analytics professionals, information services, clinical quality, and academic faculty. The primary advantage of these workgroups was the ability to bring the content and methodologic expertise a solution required for operationalization. Furthermore, by combining clinical and operational considerations for narrowly focused use cases, these workgroups were able to maintain a nimble, innovative approach to each use case.

**Figure 1:**
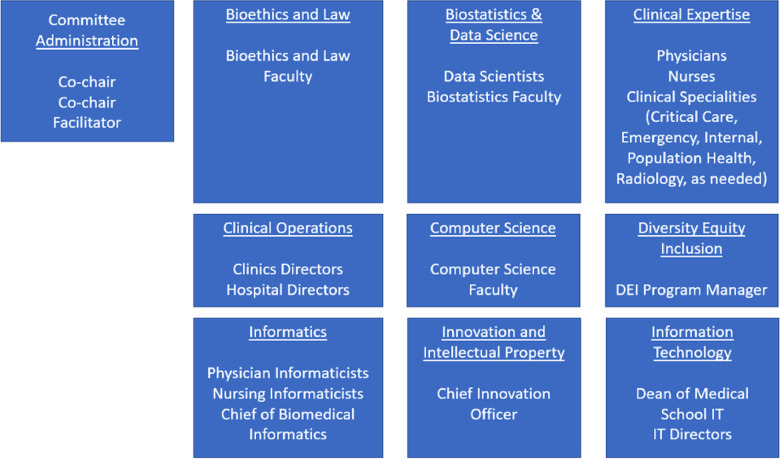
Clinical AI and predictive analytics committee composition with participants by role by respective disciplines.

**Figure 2:**
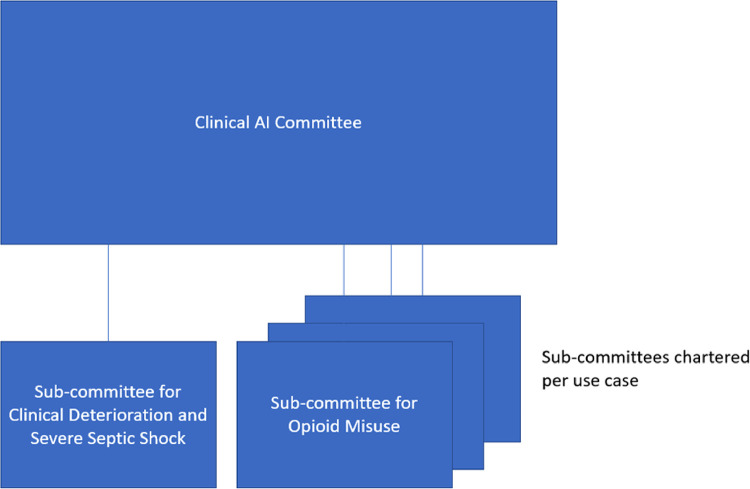
Clinical AI and predictive analytics committee and sub-committees.

However, after several solutions were enacted, weaknesses of these *ad hoc* workgroups in addressing challenges from operational and leadership domains became more apparent. From a system-wide standpoint, more integration was needed to create visibility and oversight of all models, and retain consistent governance across a variety of clinical use cases. To address these challenges while keeping the advantages of the individual use case workgroups, we created an institutional-level steering committee which would provide a front door and maintain oversight of all models while retaining individual workgroups for more detailed governance. This “Clinical AI and Predictive Analytics Committee” is multi-disciplinary and included a superset of the same disciplines that comprised the use-case specific algorithm workgroups. Figures 1, 2 show the composition and representation of the institutional committee and its relationship with the sub-committees, respectively. However, one advantage of an institutional-level committee was a stronger ethics and equity perspective. Another advantage of institution-level committee is a clear and strong connection to the University of Wisconsin campus. Connection to the campus brings academic expertise in the relevant domains, and the associated research enterprise including complementary guidelines to other institutions like the Institutional Review Board (IRB). The committee functions as a front door for the evaluation and vetting of predictive solutions prior to implementation, and for new models it commissions and oversees workgroups. The committee reports up to existing clinical and informatics leadership structures in the university and health system and provides visibility on all clinical uses of AI to these groups.

The institutional-level committee defines and establishes definitions of key terms such as “predictive model” as well as guiding principles. However, given the broad scope of potential AI applications, the committee does not seek to perform all of the duties previously performed by algorithm workgroups for each application. Rather, once an application is brought to the committee, it commissions an “Algorithm sub-committee” with the scope of a specific application. Each sub-committee follows the established guiding principles and applies them when evaluating the algorithm(s) for its specific use case, and reports back to the institutional Clinical AI and predictive Analytics Committee. This federated system seeks to retain the benefits of the application specific workgroups while realizing the advantages of a single committee to govern all applications.

### Scope

The committee oversees AI and predictive solutions which affect clinical care in the health system, including workflow and implementation. This includes solutions aimed at clinical care (e.g. patient deterioration or sepsis), patient access and resource allocation (e.g. length-of-stay (LOS) predictions, inpatient capacity management). The committee does not oversee models in which there is no clear connection to clinical care (e.g., a financial model to predict likelihood of payment). AI as a component of an FDA approved medical device is not necessarily overseen if a model isn't modifiable at the health system level and its performance has been well-characterized (e.g., an FDA approved software program to evaluate diabetic retinopathy from retinal pictures).

### Guiding principles

Below are the currently endorsed set of guiding principles:

1. Predictive model (including outside vendors or internal innovation) evaluation includes validation of performance on UW Health production data and clinician review against the appropriate target labels for application.
2. Model evaluation includes statistical measures (e.g., sensitivity, specificity, PPV) and relevant operational and health metrics (e.g., alarm rate, work-up to detection ratio, appropriate use, fairness, cost-effectiveness and intervention effectiveness on health outcomes).
3. Model output follows the five rights of Clinical Decision Support (CDS) * and is associated with interventions whenever possible.
4. Model monitoring (pilot or scale-out) includes statistical measures, operational metrics, relevant outcomes and reevaluation criteria, especially for calibration as absolute risk may change over time.
5. The basic principle of health care ethics autonomy, beneficence, justice and non-maleficence will be incorporated in all stages of model evaluation and validation. We aim to first do no harm with our AI-driven tools and ensure bioethical principles are integrated into our governance.

### Predictive solution life cycle

A key aspect of appropriate governance is establishing a full life cycle for models. This includes processes for evaluation and potential adoption of models, monitoring to ensure they continuously meet the needs of all constituents, and appropriate processes for periodic reevaluation and decommissioning of models no longer needed or functioning correctly. Given the current institutional adoption of Lean methodology and specifically A3 thinking (5), we built our approval form starting with our institutional A3 project template, but added specific questions focused on relevant questions for AI implementation. A fuller description of the usefulness of Lean's FOCUS PDCA methodology for AI can be found in our previous work (6) with a toolkit available at www.hipxchange.org/ImplementPredictiveModels. Supplementary Appendix 1 provides our model intake form, through which potential models are evaluated prior to approval. The intake form is designed to be completed in 2 stages. Basic model questions, in green, are designed to be filled out by the requestor prior to discussion with the Clinical AI and Predictive Analytics Committee. Once the committee has evaluated the use case, it can commission an algorithm subcommittee which provides the necessary expertise to complete the intake form in its entirety, which is necessary for model approval.

**Figure 3:**
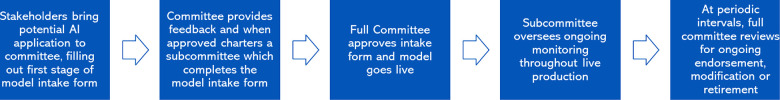
UW Health predictive model value stream.

In addition, we developed a value stream beginning with the intake form through model re-evaluation over time. Figure 3 depicts the life cycle of a typical predictive solution, from initial presentation to the Clinical AI and Predictive Analytics Committee to periodic review and update or decommissioning.

## Results

### Clear institutional apparatus for governance

At the time of this publication, the governance framework has overseen ten successful deployments, two successful retirements, and one successful non-deployment across nine applications. We expressly use the terminology of “deployment” and “retirement” as technical terms defined in the software and application development disciplines, where “deployment” refers to the promotion of the AI-driven application from a development environment into a production environment; and “retirement” is the removal of the application from a production environment after it is deemed to be no longer necessary. We distinguish this from the case of removing a solution from production due to errors or poor performance. The purpose of this technical terminology is to provide a necessary level of objectivity as it relates to endorsement, approvals, and IT change management. Applications include diverse uses of AI prediction for outputs including severe sepsis, clinical deterioration (7), physician panel weighting, COVID detection on radiographs (8), emergency department screening for falls prevention (9, 10), screening for opioid abuse (11), and Emergency Department crowding to drive adaptive staffing.

One key function of the governance framework is including all relevant AI applications. For AI applications which predated the current governance framework, there is an abbreviated process to grandfather these use cases into the current standard of oversight and transition *ad hoc* working groups to algorithm subcommittees which report up to the Committee. For AI-driven applications that are custom-built at UW Health, the University of Wisconsin, or involve a large-scale deployment, we have confidence that these are under the governance and oversight of this framework, due to the robust engagement and support of Informatics and IT within the current system. Another paradigm is the implementation of vendor-created models: these use cases are under governance particularly for vendor products that are explicitly marketed as an algorithm. However, we acknowledge that there may be use cases outside of the committee's awareness, especially for cases where the AI solution is embedded within a broader product and is not marketed as an algorithm. Finally, we note that non-clinical use cases at our institution have adopted similar principles particularly the guiding principle of local validation of model performance.

### Successful deployments spanning clinical and technology domains

We believe that one of the key drivers of our success has been comprehensive participation between clinical, operational, and IS stakeholders. While our IT professionals have a prominent role within our governance structure, AI application deployments are viewed as clinical projects analogous to other clinical initiatives in the hospital and our governance structure and use of A3 thinking mirrors that for purely clinical interventions such as clinical guideline development.

### Process for ongoing monitoring to ensure performance

Once applications have been deployed, the algorithm sub-committees continue to meet on a pre-determined frequency that is compatible with its use case and to monitor the performance of the solution over subsequent years.

We maintain that ongoing monitoring has been successful as proven by two types of occurrences. The first type that has occurred is a successive deployment where the previous version of the AI application was replaced with a more performant solution that included cases spanning a different machine learning algorithm, an updated target or prediction outcome, or a re-trained model. This necessitates that the previous solution was actively monitored and that a new solution was also evaluated and validated with clear criteria regarding performance and acceptability.

The second type of occurrences are successful retirements, where an AI solution was removed from production after the solution delivered its intended value. We wish to clearly distinguish successful retirements from successful non-deployments. The latter indicates a situation where the AI solution was deemed to be non-performant prior to its use in clinical care and was never fully deployed in the production system. This differs from a successful retirement, where the AI solution was used in production as part of routine clinical care and performing appropriately. In these cases, the needs or requirements that the solution fulfilled have changed. For example, one of our successful applications was the use of a model to predict days with high emergency department volumes at one of our hospital sites, which was used to guide a decision to call in additional physician staff. While initially useful, this model slowly became less relevant as average daily volumes increased and daily staffing was increased, obviating the need for a call-in shift or the predictive model.

### Mechanism to incorporate the equitable and ethical use of AI guidelines over time

To address the equitable and ethical use of AI, the membership of our institutional committee includes ethics expertise, including a prominent faculty member from the Law School, and staff from our office for Diversity, Equity, and Inclusion, and we maintain a line of communication with our medical bio-ethicists.

Drawing upon this expertise and membership, the guiding principles defined by the committee include guidelines for the equitable and ethical use of AI. We note that our guidelines also incorporate the evaluation of the intervention derived from the AI algorithm, which provides a more comprehensive determination of equitable and ethical impacts of how health interventions are administered across the system. Supplementary Appendix 1, the model intake form, shows explicit steps in the model vetting and monitoring process which are undertaken to ensure equity and evaluate for other potential ethical issues.

Our approach to the impactful enforcement of the equitable and ethical use of AI is to incorporate these guidelines as part of the same guiding principles that address technical and clinical guidelines. This is in contrast to treating the equity and ethics considerations as separate from other aspects of oversight. We believe this approach has been successful because it provides a clear charter for the sub-committees when applying the whole of the guiding principles to their use cases.

### Interface with research

The committee specifically oversees AI applications which are instituted by the health system for the purpose of improving clinical care. We see this as a complementary role to research oversight; AI applications for research purposes are overseen by the IRB and research leadership. The committee is made aware of research-related IT build, and works with the IRB to ensure all AI applications are governed *via* either this clinical workflow or considered research.

## Discussion

As adoption of AI applications in healthcare accelerates, there is an acute need for appropriate governance to address ethical, regulatory and trust concerns (12, 13). At the hospital level, effective governance offers the ability to specifically address these concerns while facilitating deployment and adoption (14). In our governance development, we have found that an effective system should not only be comprehensive, i.e., addressing all three domains as described in Challenges, but also adaptive, scaling appropriately with development of a program for implementing predictive solutions. This ensures that the level of oversight is proportional for each of the three domains at a given degree of program maturity. Our approach of adaptive governance evolved organically over time: we would likely have been unable to justify our current institutional committee without a number of extant solutions in need of oversight. At our current state we expect that our mechanisms will continue to evolve to meet organizational needs.

Faculty, in general have been supportive of the committee. While we expected some resistance to centralization of governance and a proscribed pathway for model deployment, these disadvantages seem to have been outweighed by the benefits of consistent expectations and process for all models. Research faculty have expressed favorable comments noting that introduces a consistent and supervised process for implementation of models after their development and validation.

While our current system developed organically, if we were to re-establish governance we would advocate a similar program of iterative building, allowing those involved in predictive model adoption to maintain flexibility early in the program and take advantage of gained institutional knowledge as it accrues. Key advantages of our current system of an oversight committee with federated small working groups include the breadth of recruited stakeholders and system scalability. Our current structure enables scaling by taking advantage of two tiers of governance. The institutional-level committee can and does charter multiple sub-committees as needed across multiple use cases to facilitate adoption and endorsement within each sub-committee's respective use cases. At the same time, the institution-level committee sets the guiding principles to enforce the consistency of standards and confidence of oversight while minimizing overhead. The goal is to meet the business needs of our health system while remaining cognizant of the AI guiding principles to prevent medical error and harm.

## Data Availability

This case study did not rely on specific data sets. Further inquiries can be directed to the corresponding authors.
