# Supplementary material for: Governance of Clinical AI applications to facilitate safe and equitable deployment in a large health system: Key elements and early successes
Source: Front Digit Health. 2022 Aug 24;4:931439. doi: 10.3389/fdgth.2022.931439 (PMC9448877; doi:10.3389/fdgth.2022.931439)
Supplement: Supplementary file 1 [file Table_1_v1.docx]

| **Model Review Form for Clinical-AI-Predictive Analytics Predictive (CAIPA)** | | |
| --- | --- | --- |
| **This form is intended to provide the Clinical-AI-Predictive Analytics (CAIPA) committee with the information needed to assess, approve, and endorse the adoption of a predictive model solution being proposed by a clinical champion. This is intended for solutions aimed at clinical care (e.g. patient deterioration or sepsis), patient access and resource allocation (e.g. length-of-stay (LOS) predictions, inpatient capacity management). This does not include financial prediction models.**  **Form content is based on established best practice recommendations for the reporting of continuous improvement and predictive model solutions^1,2,4,5^** | | |
| Sections 1-3 are required for initial submission to CAIPA and Sections 4-8 are optional with initial submission but recommended if information is known. If the model is approved for implementation at UW Health, a workgroup will be assigned that can assist with items described in sections 4-8. | | |
| Problem description/Background (please keep to 250 words) | | |
| Current Problem | Describe the problem, how the problem has been identified, why it is important to solve, when and where the problem arises, and risks of not solving this problem |  |
| Research Studies & Findings | Provide a summary of current research or literature on this problem |  |
| Reference Papers | Provide several key references to support information about the tool or solution provided above |  |
| Model Value Proposition | | |
| How will a predictive model help solve the problem | a)Explain the medical context (including whether diagnostic or prognostic) and how validation of the need for this type of model occurred  b)How has UW tried to solve this problem previously? |  |
| What is the current best practice | How do other academic medical centers handle this problem per current evidence-based practice |  |
| Are there other organizations that have models in this space that have been successful? | Briefly describe outcomes at other institutions or an indicate if this solution has not been used at other organizations to the knowledge of the submitter. |  |
| Describe how this tool could add value | Describe value as it relates to patient outcomes, safety, cost savings, efficiency and/or strategic goal alignment  How will this solution provide value over what UW is doing currently? |  |
| Describe how success of the model be evaluated | Describe the broad outcome goal i.e. increase referrals, decrease specific adverse event  Include an evaluation of statistical risk of bias, if needed here is a link to the best practice link  <http://www.probast.org/wp-content/uploads/2020/02/PROBAST_20190515.pdf> |  |
| Model Description | | |
| Algorithm type | What type of model is it? This includes basic model architecture details, such as whether it is a Naive Bayes classifier, a Convolutional Neural Network etc. |  |
| Target population | Describe the intended population of patients from which data will be drawn and the model is intended to help  (*If the intended target population differs from the patient population used in model development, please describe*) |  |
| Target outcome to be Predicted | Outcome being targeted such as likelihood of event X occurring (i.e. likelihood of sepsis in next 6 hours) |  |
| Gold Standard Definition of target outcome | Provide information on how the outcome will be identified in practice. For example, CMS definition of sepsis, ICD-10 code, discrete documentation in EHR etc. and whether any data will be used as short-term proxy for true outcome of interest. |  |
| Missing data strategy (if known) | Describe how missing data was addressed, imputed, or corrected during model development and whether strategy might differ for implementation |  |
| Features List | List the variables that will be used in model as inputs and include data type if known (nominal, categorical, dichotomous, continuous etc.) |  |
| **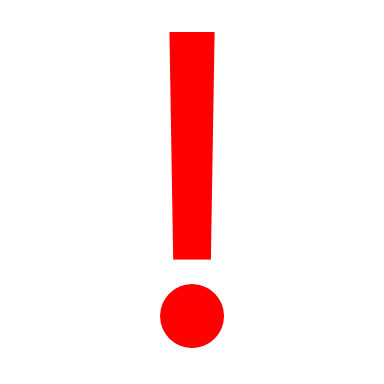The following sections are optional with initial submission but recommended if information is known** | | |
| Model Information | | |
| License information | If vendor or other proprietary model, please describe license information |  |
| Expert Contact | Contact information for model developer or content expert on the model |  |
| Transparency | Describe whether model code and development data are available to the community to review |  |
| Version Control (if applicable) | What version of the model is it and how does it differ from previous version (if applicable) |  |
| Proposed workflow integration | | |
| Intended use | Describe the task the model is intended to perform, i.e. clinical decision support, resource allocation.  Describe the organizational engagement on this workflow currently |  |
| Model output | Describe the computed result of the model i.e. low/high risk, yes/no, % liklihood, and/or model explainers such as feature importance or contributing factors |  |
| Target User | Describe the intended user role(s) of the model output (MD, nurse, clinic manager etc.) |  |
| EHR or other integration point | Modality for integration into decision support and/or workflows i.e. Dashboard, icon, BPA, paging |  |
| Scoring timeframe and context | Frequency of model evaluation of patient data and other workflow activity needed to trigger evaluation by the model i.e. models evaluates data every 15 minutes or model evaluates patient at time of discharge |  |
| Out of scope use | Describe any patient populations or clinical settings where the use of this model would not be appropriate |  |
| Ethical considerations | Describe any potential issues related to performance of the model for different groups. For example, if there is a risk that this model could work better for some groups than others or otherwise lead to unjust or prejudicial treatment.  **if you need some guidance on how to perform this assessment please review this (*[*link*](https://www.raceforward.org/sites/default/files/RacialJusticeImpactAssessment_v5.pdf)*) from Examining the Cause” equity questions from Race Forward’s Racial Equity Impact Assessment (REIA)* |  |
| Validation on UW Health Data | | |
| Description of local dataset | Description of dataset (number of patients, demographics of patients, timeframe, variables etc.) |  |
| Mapping to EHR | Describe verification strategy of Cross-Mapping Features to Production Data |  |
| Equity Analysis | Describe assessments to test for unequal performance across groups |  |
| Performance | Describe performance of model i.e. AUC, Confusion Matrix, Positive Predictive Value etc. |  |
| Decision Thresholds | Describe decision threshold(s) and rationale for choice of threshold(s) |  |
| Monitoring Strategy | | |
| Frequency |  |  |
| Process and outcome measures |  |  |
| Clinical Safety Review |  |  |
| Other | Please provide additional information about the model not covered above if applicable  Examples : Has there been any consideration to the possibility of commercializing ? |  |

**Glossary of Terms^3,6^**

**ACCURACY (error rate)** The rate of correct (incorrect) predictions made by the model over a data set (cf. coverage). Accuracy is usually estimated by using an independent test set that was not used at any time during the learning process. More complex accuracy estimation techniques, such as cross-validation and the bootstrap, are commonly used, especially with data sets containing a small number of instances.

**ALGORITHM** A set of step-by-step instructions. Computer algorithms can be simple (if it's 3 p.m., send a reminder) or complex (identify pedestrians).

**ATTRIBUTE (field, variable, feature)** A quantity describing an instance. An attribute has a domain defined by the attribute type, which denotes the values that can be taken by an attribute. The following domain types are common:

**Categorical** A finite number of discrete values. The type nominal denotes that there is no ordering between the values, such as last names and colors. The type ordinal denotes that there is an ordering, such as in an attribute taking on the values low, medium, or high.

**Continuous** (quantitative) Commonly, subset of real numbers, where there is a measurable difference between the possible values. Integers are usually treated as continuous in practical problems.

A **feature** is the specification of an attribute and its value. For example, color is an attribute. ``Color is blue'' is a feature of an example. Many transformations to the attribute set leave the feature set unchanged (for example, regrouping attribute values or transforming multi-valued attributes to binary attributes). Some authors use feature as a synonym for attribute (e.g., in feature-subset selection)

**BLACK BOX** A description of some deep learning systems. They take an input and provide an output, but the calculations that occur in between are not easy for humans to interpret.

**CLASSIFIER** A mapping from unlabeled instances to (discrete) classes. Classifiers have a form (e.g., decision tree) plus an interpretation procedure (including how to handle unknowns, etc.). Some classifiers also provide probability estimates (scores), which can be thresholded to yield a discrete class decision thereby taking into account a utility function.

**CONFUSION MATRIX** A matrix showing the predicted and actual classifications. A confusion matrix is of size LxL, where L is the number of different label values. The following confusion matrix is for L=2:

| actual \ predicted | Negative | Positive |
| --- | --- | --- |
| Negative | a | b |
| Positive | c | d |

The following terms are defined for a two by two confusion matrix: Accuracy (a+d)/(a+b+c+d). True positive rate (Recall, Sensitivity) d/(c+d). True negative rate (Specificity) a/(a+b). Precision d/(b+d). False positive rate b/(a+b). False negative rate c/(c+d).

**CROSS VALIDATION** A method for estimating the accuracy (or error) of an inducer by dividing the data into k mutually exclusive subsets (the ``folds'') of approximately equal size. The inducer is trained and tested k times. Each time it is trained on the data set minus a fold and tested on that fold. The accuracy estimate is the average accuracy for the k folds.

**DECISION THRESHOLD** A decision threshold is a value that dichotomizes the result of a quantitative test to a simple binary decision. The test result of a quantitative diagnostic test is dichotomized by treating the values above or equal to a threshold as positive, and those below as negative, or vice-versa.

**DEEP LEARNING** How a neural network with multiple layers becomes sensitive to progressively more abstract patterns. In parsing a photo, layers might respond first to edges, then paws, then dogs

**EXPERT SYSTEM** A form of AI that attempts to replicate a human's expertise in an area, such as medical diagnosis. It combines a knowledge base with a set of hand-coded rules for applying that knowledge. Machine-learning techniques are increasingly replacing hand coding.

**MACHINE LEARNING** The use of algorithms that find patterns in data without explicit instruction. A system might learn how to associate features of inputs such as images with outputs such as labels.

**MISSING VALUE** The value for an attribute is not known or does not exist. There are several possible reasons for a value to be missing, such as: it was not measured; there was an instrument malfunction; the attribute does not apply, or the attribute's value cannot be known. Some algorithms have problems dealing with missing values.

**MODEL** A structure and corresponding interpretation that summarizes or partially summarizes a set of data, for description or prediction. Most inductive algorithms generate models that can then be used as classifiers, as regressors, as patterns for human consumption, and/or as input to subsequent stages of the Knowledge Discovery process.

**PREDICTIVE MODEL** – an algorithm created using train and test data to make a prediction for diagnostic, prognostic, or treatment purposes.

**REINFORCEMENT LEARNING** A type of machine learning in which the algorithm learns by acting toward an abstract goal, such as “earn a high video game score” or “manage a factory efficiently.” During training, each effort is evaluated based on its contribution toward the goal.

**SUPERVISED LEARNING** A type of machine learning in which the algorithm compares its outputs with the correct outputs during training. In unsupervised learning, the algorithm merely looks for patterns in a set of data.

**UNSUPERVISED LEARNING** Learning techniques that group instances without a pre-specified dependent attribute. Clustering algorithms are usually unsupervised.

**UW HEALTH PREDICTIVE MODEL** A predictive model *performant on UW Health’s own data*

**UW HEALTH PREDICTIVE SOLUTION** UWH Health Predictive model and the delivery mechanism and workflows directly associated with the model output

**References**

1. A3 Process: <https://uconnect.wisc.edu/inside-uw-health/mission-watch/uw-health-way/toolkit/tools/a3/>
2. Collins, G. S., Reitsma, J. B., Altman, D. G., & Moons, K. G. (2015). Transparent Reporting of a Multivariable Prediction Model for Individual Prognosis or Diagnosis (TRIPOD) The TRIPOD Statement. Circulation, 131(2), 211-219Hernandez-Boussard, T., Bozkurt, S., Ioannidis, J. P., & Shah, N. H. (2020).
3. Hutson, M. (2017). AI Glossary: Artificial intelligence, in so many words. *Science*, *357*(6346), 19-19.
4. MINIMAR (MINimum Information for Medical AI Reporting): developing reporting standards for artificial intelligence in health care. *Journal of the American Medical Informatics Association*, *27*(12), 2011-2015.
5. Mitchell, M., Wu, S., Zaldivar, A., Barnes, P., Vasserman, L., Hutchinson, B., ... & Gebru, T. (2019, January). Model cards for model reporting. In *Proceedings of the conference on fairness, accountability, and transparency* (pp. 220-229).
6. Provost, F., & Kohavi, R. (1998). Glossary of terms. *Journal of Machine Learning*, *30*(2-3), 271-274.
